# Developmental Maturation and Alpha-1 Adrenergic Receptors-Mediated Gene Expression Changes in Ovine Middle Cerebral Arteries

**DOI:** 10.1038/s41598-018-20210-w

**Published:** 2018-01-29

**Authors:** Dipali Goyal, Ravi Goyal

**Affiliations:** Center for Perinatal Biology, School of Medicine, Loma Linda University, Loma Linda, CA USA

## Abstract

The Alpha Adrenergic Signaling Pathway is one of the chief regulators of cerebrovascular tone and cerebral blood flow (CBF), mediating its effects in the arteries through alpha1-adrenergic receptors (Alpha1AR). In the ovine middle cerebral artery (MCA), with development from a fetus to an adult, others and we have shown that Alpha1AR play a key role in contractile responses, vascular development, remodeling, and angiogenesis. Importantly, Alpha1AR play a significant role in CBF autoregulation, which is incompletely developed in a premature fetus as compared to a near-term fetus. However, the mechanistic pathways are not completely known. Thus, we tested the hypothesis that as a function of maturation and in response to Alpha1AR stimulation there is a differential gene expression in the ovine MCA. We conducted microarray analysis on transcripts from MCAs of premature fetuses (96-day), near-term fetuses (145-day), newborn lambs, and non-pregnant adult sheep (2-year) following stimulation of Alpha1AR with phenylephrine (a specific agonist). We observed several genes which belonged to pro-inflammatory and vascular development/angiogenesis pathway significantly altered in all of the four age groups. We also observed age-specific changes in gene expression–mediated by Alpha1AR stimulation in the different developmental age groups. These findings imply complex regulatory mechanisms of cerebrovascular development.

## Introduction

The brain is an organ of high metabolic demand that at rest consumes ~20% of the body’s oxygen (O_2_), despite comprising only ~2% of body weight^1^. Of critical importance, in response to many stressors both immature animals and adults are subjected to dysregulation of cerebral blood flow (CBF) and function. Nonetheless, the cellular and molecular mechanisms by which the cerebrovasculature develops and is regulated remain enigmatic; this is of particular importance in terms of cerebral pathophysiology. For instance, the premature fetus, near-term fetus, and newborn infant, in response to CBF dysregulation, may develop hypoxic-ischemic encephalopathy (HIE), germinal matrix hemorrhage, intracerebral hemorrhage, intraventricular hemorrhage, and related problems^2–6^. In turn, these pathologies may give rise to clinical pathologies such as cerebral palsy, mental retardation, behavioral disorders, and other sequelae with long-lasting medical and socioeconomic consequences^2,7^. A sobering reality is that despite the extensive application of fetus heart rate monitoring for the detection of fetus asphyxia and the rising rate of cesarean section, there has been no diminution in the rates of cerebrovasculature dysfunction –mediated complications^8,9^.

Of importance, norepinephrine is one of the chief regulators of cerebral vasculature during development as well as in disease and stress^10–13^. Norepinephrine is a well-studied stress hormone and a major chemical messenger of adrenergic nervous system^14–16^. Importantly, the cerebral vasculature is richly innervated, the largest component of which is adrenergic in nature^17–19^, and is thought to serve both vasomotor and trophic functions^20–22^. Despite recognition of cerebral adrenergic innervation for more than a century, there has remained some controversy about the regulatory role adrenergic nerves play in the brain^23^. Some have argued that the sympathetic system has a little biological significance in cerebral vasculature^24,25^. In contrast, evidence, particularly in a developing organism, supports a dynamic role in cerebrovascular autoregulation in response to changes in blood pressure^26–29^, and in the maintenance of cerebrovascular tone^30^. An important barrier towards the study of the role of norepinephrine was its inability to cross the blood-brain barrier^16^. Other reasons which may play a role in the confusion were the species differences. While in the cat an abrupt rise in arterial pressure produced a transient increase in CBF that is attenuated by stimulation of the cervical sympathetic nerves (adrenergic stimulation), this was not seen in the dog^31^. However, the evidence demonstrates that developmental age is an important determinant of cerebrovascular response to sympathetic stimulation, fetuses and newborns being particularly sensitive in this regard. Active cerebrovascular autoregulation was demonstrated in the newborn dog pup^32^ and piglets^33,34^, and in near-term fetus lambs^35,36^, as well as in preterm lambs^37^.

The major receptor which mediates norepinephrine’s vascular effect is Alpha-1 Adrenergic Receptors (Alpha1AR)^28,38^. In tracheotomized, pancuronium bromide paralyzed lambs (<2 weeks of age) electrical stimulation of the superior cervical ganglion (SCG; sympathetic ganglion) reduced ipsilateral CBF by 25 ± 3%. In contrast, following Alpha1AR inhibition by the antagonist prazosin this decrease was only 5 ± 1%^39^. Thus, it is apparent that Alpha1AR plays a critical role in CBF autoregulation. Moreover, with increasing age the anesthetized preterm lamb fetus (94 to 121 days’ gestation), term lambs (127 to 140 days), newborn lambs (7 to 14 days) and adult sheep prepared with a cranial window showed a significant decrease in pial arterial response to infused norepinephrine^40^. Importantly, norepinephrine acts postsynaptically on smooth muscle adrenergic receptors as a determinant of vascular contractility^41–43^. Mechanistic pathways are not completely understood, however.

In studies on adult humans, CBF increased following SCG blockade^29,44,45^. Moreover, in human, studies have demonstrated a significant reduction in cerebral oxygenation following stimulation with norepinephrine as well as phenylephrine^46–48^. Similar to these studies, in sheep we observed significant contractile responses following stimulation with norepinephrine and phenylephrine which were reversed by Alpha1AR antagonist^38^. Similarly, in a term newborn lambs, removal of SCG was associated with linear increase in CBF with increase in mean arterial blood pressure. However, with intact SCG innervation, when mean arterial blood pressure was increased, CBF and O2 consumption remained constant^26^. In contrast, in a preterm lamb, even with intact SCG, CBF increased linearly with increase in mean arterial blood pressure indicating that sympathetic system in not fully mature at this stage^26^. Furthermore, others and we have reported that cerebrovascular reactivity, including that of alpha1-adrenergic-mechanisms, change dramatically with maturational development^26,38,42,43,49^. Also, we have demonstrated that there is a significant increase in Alpha1AR expression with maturation^38^. Importantly, other than regulation of CBF, Alpha1AR has been demonstrated to play a critical role in angiogenesis^21,22^. However, the underlying genetic pathways/networks regulated by Alpha1AR are not well studied. Since their development, microarrays have proven to be powerful tools in the elucidation of gene expression patterns, in response to various physiological and pathological variables. Treatment with Alpha1AR specific agonist phenylephrine (PHE) is been reported with rapid induction of gene expression in the rat neonatal myocardial cells^50^. We have also reported the gene expression profile of sheep carotid artery with reference to maturational development^51^. In continuation to our earlier study, we tested the hypothesis that as a function of developmental maturation and in response to Alpha1AR stimulation there is a differential gene expression in the ovine middle cerebral artery (MCA).

## Materials and Methods

### Experimental animals and tissues

All experimental procedures were performed within the regulations of the Animal Welfare Act, the National Institutes of Health Guide for the Care and Use of Laboratory Animals, the Guidelines of the American Physiological Society, and were approved by the Animal Care and Use Committee of Loma Linda University. For these studies, we used cerebral arteries from premature fetuses (gestational age - 96-days old), near-term fetuses (gestational age 140 days), newborn lambs (5 days old) and non-pregnant adult sheep (18–24 months) obtained from Nebeker Ranch (Lancaster, CA). For each experiment, four animals were used; in case of fetus twins only one of the twins was included in the study. In our previous studies, we have described the isolation of MCA from sheep^38,52,53^. Briefly, pregnant and non-pregnant ewes were anesthetized with thiopental sodium (10 mg/kg, Intravenous) and anesthesia was maintained with inhalation of 1% of isoflurane in oxygen throughout surgery. Following the delivery of the fetus by hysterotomy, fetuses and ewes were euthanized with an overdose of proprietary euthanasia solution, Euthasol (pentobarbital sodium 100 mg/kg and phenytoin sodium 10 mg/kg; Virbac, Ft. Worth, TX). Studies were performed in isolated MCAs cleaned of adipose and connective tissue. From each animal, cleaned MCA were cut into segments ∼4 mm length, and placed in tissue baths containing Krebs buffer (containing in mM: 120 NaCl; 4.8 KCl; 1.2 K_2_HPO_4_; 25 NaHCO_3_; 1.2 MgCl_2_; 2.5 CaCl_2_ and 10 glucose) bubbled with 95% O_2_/5% CO_2_ for 30 minutes. We exposed the arteries to PHE (10 µM) at 38 °C, the contraction of the arteries was monitored and following exposure for 30 minutes arterial segments were snap frozen in the organ bath and stored in −80 degrees for RNA isolation.

Microarray analysis was conducted by utilizing the commercial services of GenUs Biosystems (Northbrook, Illinois). In previous studies, we have described this technique in detail^2–4^. Briefly, MCA segments were homogenized and lysed in Trizol (Ambion, Austin, TX) and total RNA was isolated using standard procedure followed by purification over spin columns (Ambion). Nanodrop 2000 Spectrophotometer was used to measure total RNA concentration and purity at optical density 260 and 280. Quality and degradation were assessed using an Agilent Bioanalyzer with RNA 6000 Nano Lab Chip (Agilent Technologies, Santa Clara, CA). RNA with integrity number >8 were used for preparing labeled cRNA by amplifying the Poly(A) + RNA population within the total RNA sample. About 1 µg of total RNA was primed with a DNA oligonucleotide containing the T7 RNA polymerase promoter 5′ to a d(T)24 sequence and reverse transcribed. Following second-strand cDNA synthesis and purification of double-stranded cDNA, *in vitro* transcription was performed using T7 RNA polymerase. Labeled cRNA quality and quantity was assayed by spectrophotometry and Agilent Bioanalyzer. One µg of purified cRNA was fragmented to uniform size and applied to Agilent Sheep Gene Expression Microarray, 8 × 15 K (Design ID 019921, Agilent Technologies) in hybridization buffer. Hybridization of the arrays was conducted at 65 °C for 17 hours in a shaking incubator. Following hybridization, the array chips were washed at 37 °C for 1 min, then rinsed and dried. Following this the array chips were scanned at 5 µm resolution with an Agilent G2565 Microarray Scanner (Agilent Technologies). The scanned images were processed with Agilent Feature Extraction software and the data generated for each probe on the array was analyzed with GeneSpring GX v7.3.1 software (Agilent Technologies). Annotations are based on the Agilent eArray annotation file dated January 2010.

### Real-time PCR validation

Microarray analysis results were validated by real-time PCR. We chose the top 6 upregulated and 6 downregulated genes for analysis using real time PCR. Primer 3 web-based software (http://frodo.wi.mit.edu/primer3/) was used to design the primers using the same probe sequences as those on the microarray chip. The primers were synthesized by Integrated DNA technologies (Coralville, CA). Total RNA (1 ug per reaction) was reverse transcribed using a Quantitect reverse transcriptase kit (Qiagen, Valencia, CA). Relative expression was normalized to 18 S RNA and fold-changes were calculated using the ΔΔ cycle threshold (CT) method^5^. Samples were analyzed on the Qiagen RotorGene Real-Time PCR machine. Following the validation of the microarray data, downstream differential gene expression, pathway/network analysis, and upstream regulator analysis was conducted.

### Pathway/Network Analysis

We have described these methods in our previous publications^51,54–56^. We analyzed the annotated genes using Ingenuity Pathway Analysis Program (Ingenuity Systems, Redwood City, CA). The pathways identified were ranked based on the ratio of the number of molecules in a given pathway that are altered in the present dataset versus the total known molecules that constitute the pathways. The p-value of the association between specific canonical pathway and the genes present in the test dataset was calculated using Fisher’s Exact Test (P value < 0.05 was considered significant). The ratio was used to measure the number of genes overlap and p-value to measure the confidence of association. Z-score was also calculated to examine the activation or inhibition status of a particular pathway in the present dataset. For the calculation of Z-score, gene expression from Ingenuity knowledge base (published literature) was compared with gene expression changes observed in the present dataset. For example, if the activation of a particular pathway was associated with upregulation of a gene in a particular canonical pathway in the knowledge base and the present dataset it was assigned a score of 1. Similarly, if the activation of a particular pathway was associated with the downregulation of a gene in a particular canonical pathway in both knowledge base and the present dataset it was assigned the score of 1. However, if the activation of a particular pathway was associated with a change in gene expression which is opposite of the observed change in the present dataset it was assigned a score of −1. Finally, all the genes which belonged to a particular pathway were examined, and a total score was assigned. Z-score of >2 or <−2 was considered significant.

### Upstream Regulator Analysis

We have described these methods in our previous publications^51,54–56^.The goal of the upstream regulator analysis was to identify the signal transduction regulators that can mimic the observed gene expression changes in the present dataset with respect to the biological activities occurring in the tissues or cell system. Upstream regulator analysis was conducted using IPA software. The direction of change in the gene expression observed in the experimental samples (relative to a control) was compared for changes in gene expression observed by application of a particular upstream regulator as published in the literature. Each potential upstream regulator was analyzed by using two statistical measures: an overlap p-value and an activation z-score^2,27^. The overlap p-value was based on significant overlap between dataset genes and known targets regulated by an upstream regulator. The activation z-score was used to infer activation states of upstream regulators based on comparison with a model that assigns random regulatory directions (http://ingenuity.force.com/ipa/IPATutorials?id=kA250000000TNF7CAO).

### Statistics

Individual expression values across arrays were compared by normalizing raw intensity data from each gene to the 75th percentile intensity of each array. Only genes with values greater than background intensity for all samples within each group were used for further analysis. Differentially expressed genes were identified by 1.5-fold change and Welch T-test p-values < 0.05 between each age or vessel group. Statistical significance in the real-time PCR data was determined by one-way analysis of variance (ANOVA) and post-hoc Newmans-Keul test.

## Results

**Figure 1:**
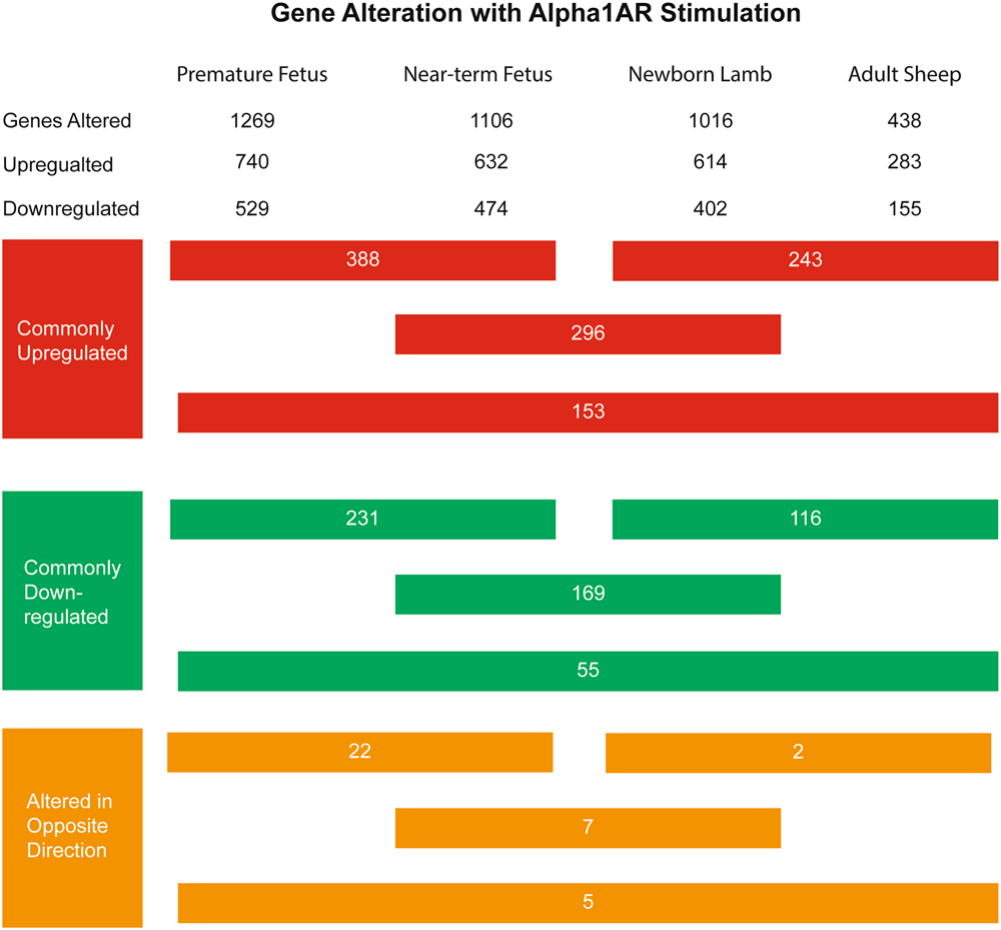
Flowchart demonstrates the number of genes altered in middle cerebral arteries at different developmental ages.

**Figure 2:**
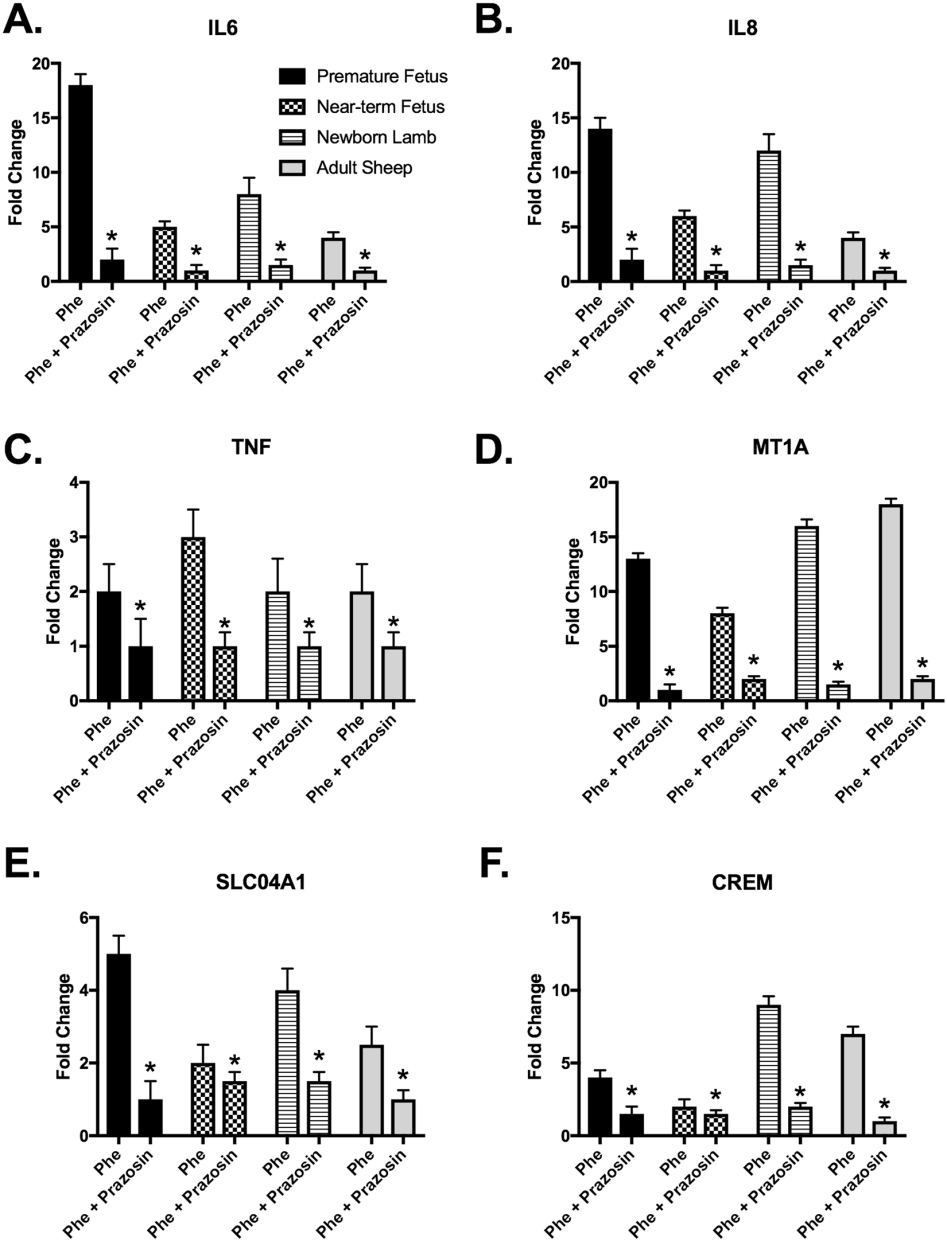
Bar graphs demonstrating mRNA expression of upregulated genes by quantitative real-time PCR in response to Alpha1AR specific agonist phenylephrine (PHE) and Alpha1AR specific antagonist (Prazosin). N = 4 for each experiment and * denotes P < 0.05.

**Figure 3:**
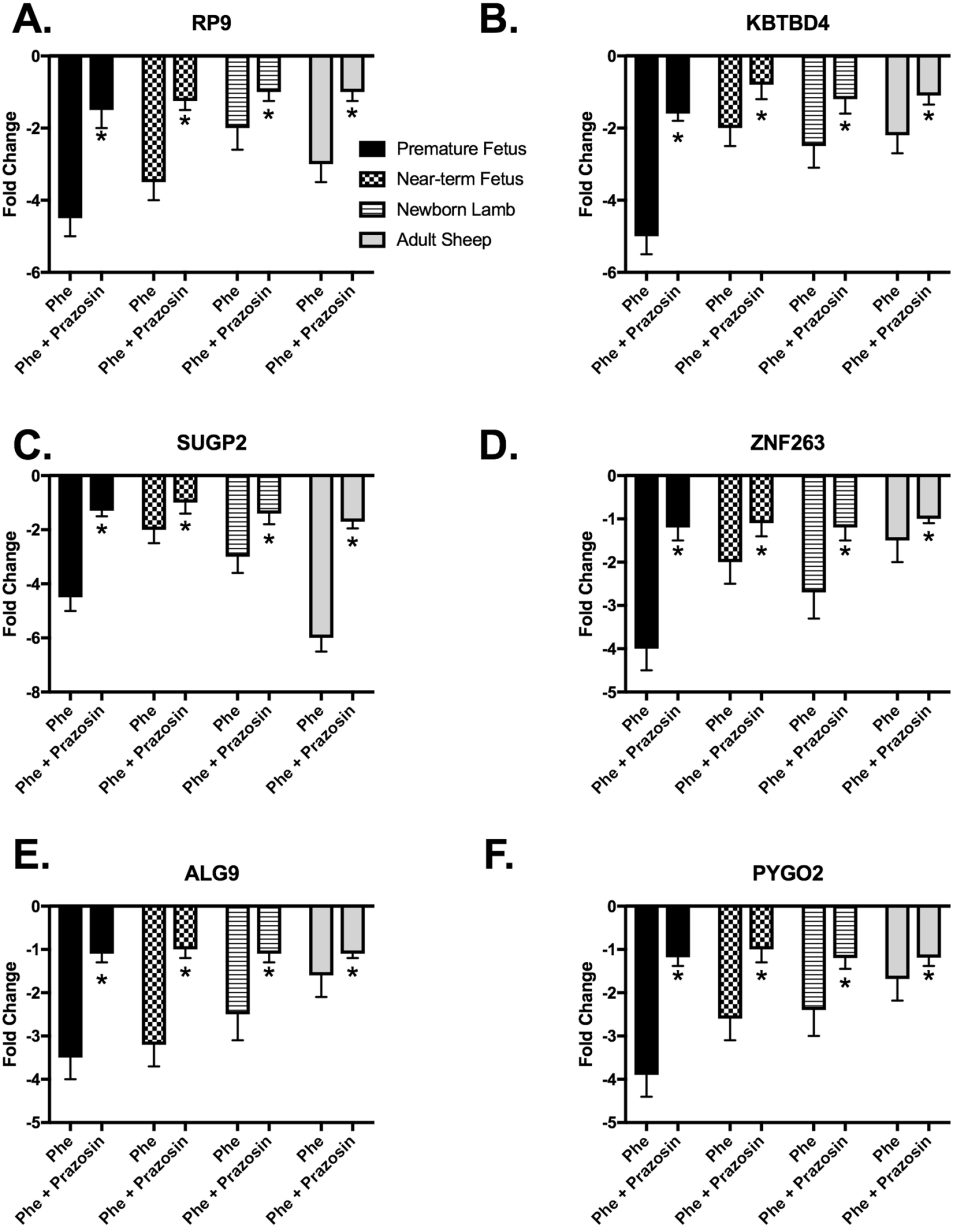
Bar graphs demonstrating mRNA expression of downregulated genes by quantitative real-time PCR in response to Alpha1AR specific agonist phenylephrine (PHE) and Alpha1AR specific antagonist (Prazosin). N = 4 for each experiment and * denotes P < 0.05.

Stimulation of arterial segments with PHE produced strong contractile responses in all the age groups studied. To confirm the response to PHE is mediated by Alpha1AR, we added prazosin (Alpha1AR specific antagonist) and observed complete inhibition of PHE-induced contractile response. As we have published this finding in the past, the results are not included in the present manuscript^38,57,58^. Following exposure to PHE, we conducted transcriptomic expression analysis and observed a significant difference in the expression profile with PHE treatment in all the four groups (Fig. 1). To further validate the findings that PHE treatment induced gene expression was in response to Alpha1AR activation, we blocked the receptors by application of prazosin. We observed inhibition of PHE-induced alteration in gene expression changes in top 6 upregulated and top 6 downregulated genes as observed in microarray experiments (Figs 2 and 3). Of interest, we observed that the number of genes altered with PHE treatment reduced with increasing age. The premature fetus lamb MCA had ~300% higher genes altered as compared to those in adults MCA (Fig. 1). Next, we compared the genes which were commonly altered in the different age groups and observed a significant overlap in altered gene expression on stimulation with Alpha1AR in different age groups (Fig. 1). Original data sets and raw files are uploaded to GEO database (Accession no. GSE109325). The genes, pathways, and upstream regulators altered by Alpha1AR stimulation in MCA obtained from premature fetuses, near-term fetuses, newborn lambs, and adult sheep are provided in Supplemental Tables S1, S2, and S3, respectively.

### Alpha1AR–mediated gene expression in the ovine MCA

**Figure 4:**
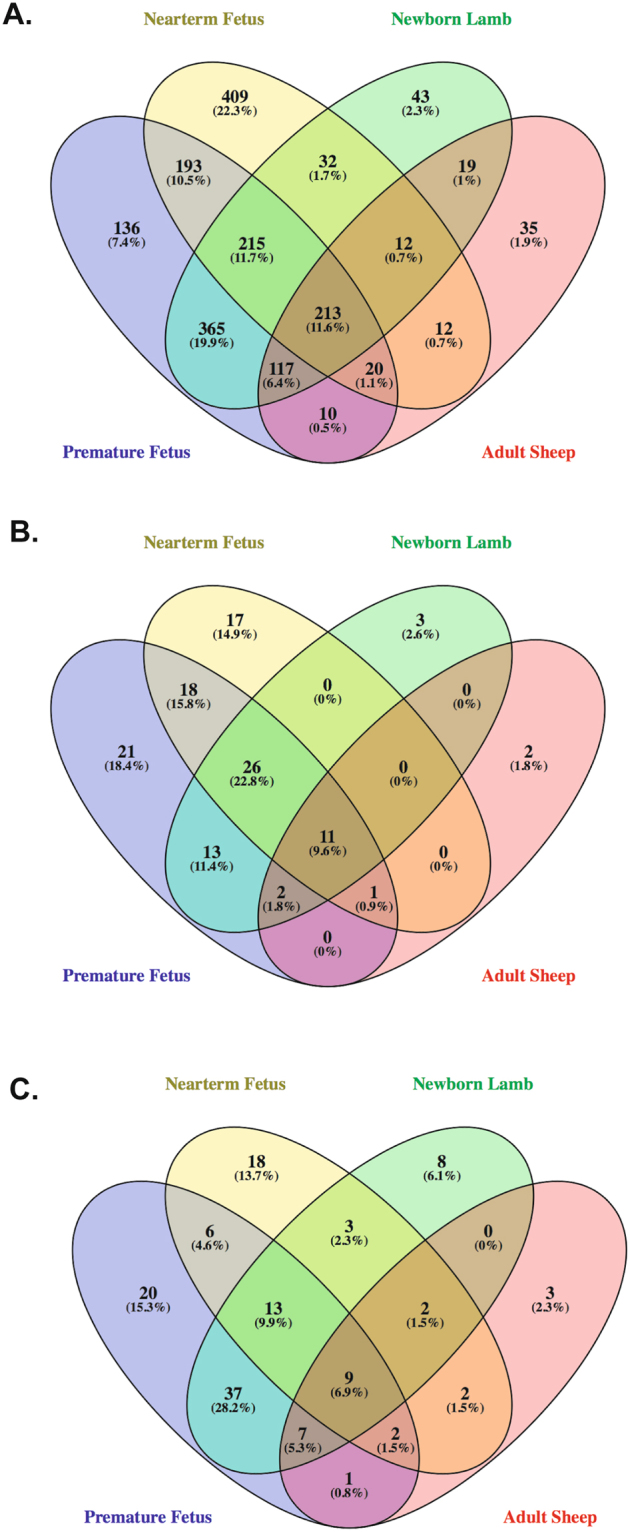
Venn diagram demonstrating the number of (**A**) genes altered (**B**) pathways altered and (**C**) upstream regulators altered in response to Alpha1AR activation in middle cerebral arterial segments from sheep at different developmental age groups.

**Figure 5:**
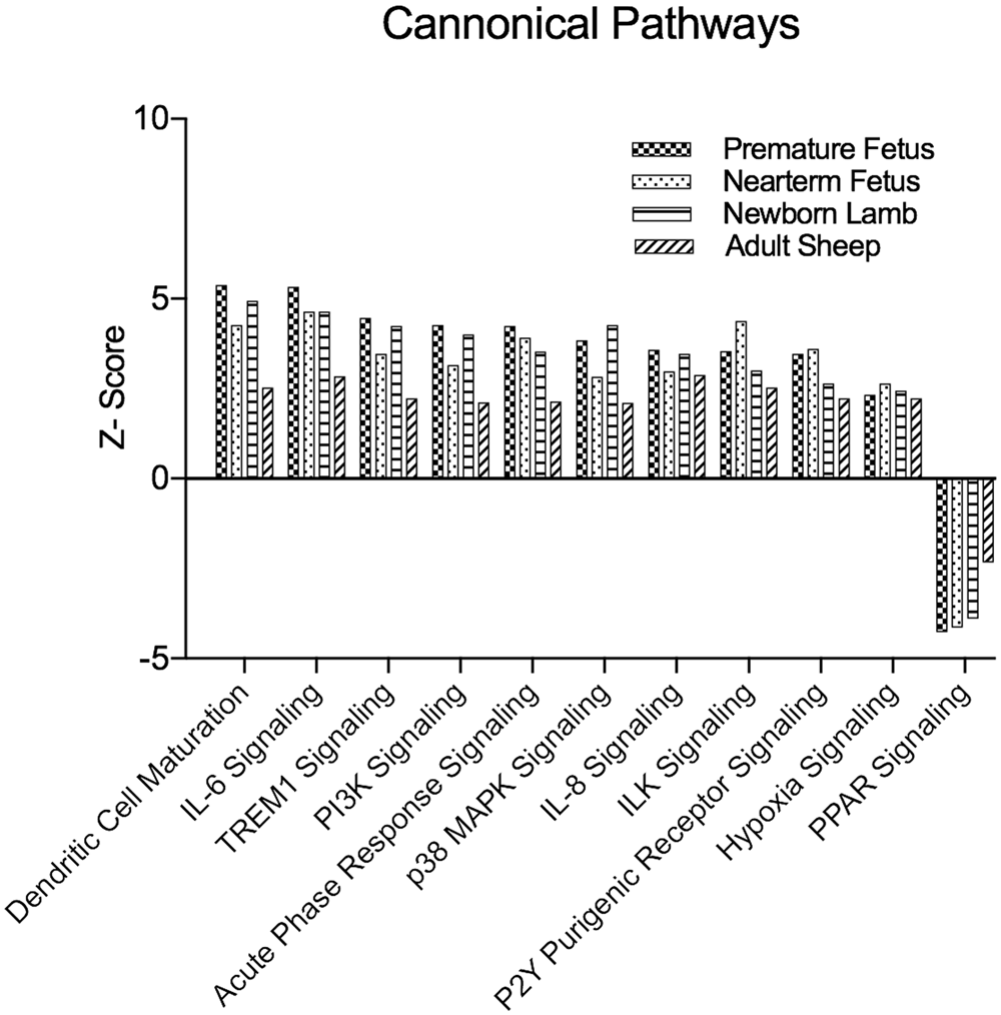
Bar graph demonstrating canonical pathways commonly altered in response to Alpha1AR activation in middle cerebral arterial segments from all four developmental age group studied.

PHE-mediated stimulation of Alpha1AR leads to alteration of 213 genes commonly in all the four groups irrespective of the maturational age (Fig. 4A). Of these 119 were upregulated and 55 were downregulated in all developmental stages on stimulation of Alpha1AR. Specific genes are provided in Supplementary Table S4. On conducting pathway analysis, we identified 10 pathways commonly activated and 1 pathway inactivated in all the developmental ages in response to Alpha1AR stimulation (Fig. 4B). These were dendritic cell maturation, IL6 signaling, IL8 signaling, ILK signaling, TREM1 signaling, PI3K signaling, acute phase response signaling, p38 MAPK signaling, P2Y purinergic receptor signaling, and Hypoxia signaling. The only inactivated pathway common to all groups was PPAR signaling (Fig. 5). Overall, the genes involved in the inflammatory response were upregulated in all the four group in response to Alpha1AR stimulation. On examining the upstream regulators altered by Alpha1AR stimulation of MCA irrespective of the developmental age, we identified 9 upstream regulators in the activated state; these were TNF, IL6, IKBKB kinase, FOXO1, PTGS2, EGR1, SELP, KLF4, and NFKBIA. All these were significantly upregulated following PHE stimulation in all the age group studied (Fig. 4C).

### Differential gene expression in response to Alpha1AR stimulation in the MCA from premature fetuses versus near-term fetuses

**Figure 6:**
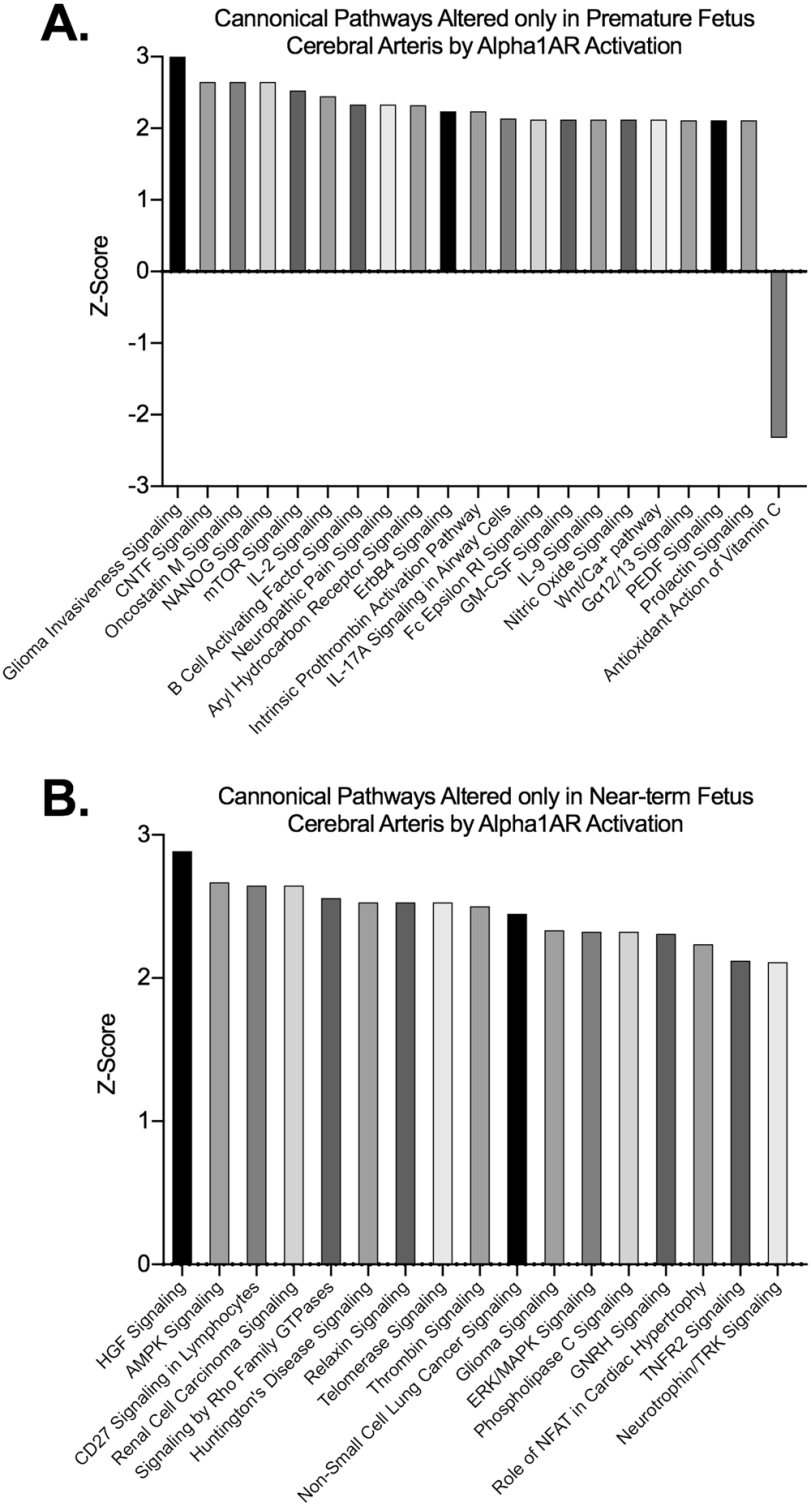
Bar graphs demonstrating canonical pathways altered in response to Alpha1AR activation in middle cerebral arterial segments exclusively in (**A**) premature fetus and (**B**) near-term fetus.

Others and our previous research have demonstrated that the adrenergic regulation is not fully developed in premature fetuses. To examine the changes in Alpha1AR stimulation mediated changes in gene expression with fetal maturation, we determined the differential expression of genes between the two groups. As demonstrated in Table 1, we observed that 1269 genes were altered in the premature fetus MCA as compared to 1106 genes in the near-term fetus MCA. Of these, 641 genes were commonly altered in both premature fetuses and near-term fetuses; 388 genes were upregulated in both age groups and 231 were downregulated (Supplementary Table S5). Importantly, 22 genes were altered in opposite direction with maturation from preterm to near-term fetuses. We further identified the genes which were only altered in the premature fetus but not in the near-term fetus MCA on Alpha1AR stimulation (Supplementary Table S5). We observed 629 genes altered exclusively in the premature fetus MCA, out of these 344 genes were upregulated and 284 were downregulated (Supplementary Table S5). Further, with maturation, there was >1.5-fold upregulation of 232 genes and downregulation of 234 genes in the near-term fetus MCA (Supplementary Table S5). Next, we conducted pathway analysis on the genes which were altered exclusively in the premature fetus and not in the near-term fetus. A total of 33 pathways were altered exclusively in the premature fetus (and not in the near-term fetus). Of these, 31 were activated and 3 were inactivated (Supplementary Table S6). Moreover, on comparing with all the other developmental stages, we identified 20 pathways exclusively activated by Alpha1AR in premature fetus cerebral arteries and one pathway inactivated (Fig. 6). The top three pathways activated were Tec Kinase Signaling, iCOS-iCOSL Signaling, and PKCθ Signaling. The 3 inactivated signaling pathways were LXR/RXR Activation, PPARα/RXRα Activation, and Antioxidant Action of Vitamin C pathway. Similarly, we examined the pathways which were altered only in the near-term fetus and not in a premature fetus MCAs. We identified 17 pathways which were activated in a near-term fetus which were not activated at the premature stage (96-day old fetus). None of the pathways was found to be inactivated in the near-term fetus as compared to the premature fetus (Supplementary Table S6). The top three pathways which were only altered in the near-term fetus MCA were HGF signaling, AMPK signaling, and CD27 Signaling. We also examined the pathways which were altered in both the premature and the near-term fetus in response to Alpha1AR stimulation. A total of 58 pathways were altered of which 57 were activated and one pathway was inactivated on stimulation with Alpha1AR at both developmental stages (Supplementary Table S6). The top three commonly altered pathways were IL-6 signaling pathway, ILK signaling pathway, and Dendritic Cell Maturation pathway. Next, we conducted upstream regulator analysis, to identify molecules commonly altered by Alpha1AR stimulation in both the premature fetus and the near-term fetus MCA. We identified 29 upstream regulators activated and 1 upstream regulator inactivated by Alpha1AR stimulation in both the premature fetus and the near-term fetus MCA (Supplementary Table S7). The top three activated upstream regulators were TNF, IL1B, and IL6. The three inhibited upstream regulators were ZFP36, DUSP1, and KLF2 signaling molecules. Of note, on comparing the upstream regulators in the premature fetus versus the near-term fetus, we identified 52 regulators to be activated and 13 regulators to be in the inactivated state only in premature fetus MCA on stimulation with Alpha1AR agonist and not in those from the near-term fetus (Supplementary Table S7). Similarly, we identified 17 upstream regulators activated and 8 upstream regulators inactivated exclusively in the near-term fetus MCA on stimulation with Alpha1AR agonist as compared to MCA from the premature fetus (Supplementary Table S7). The top three upstream regulators which were exclusively altered with the maturation of fetus MCA were TGFB3, HMGBI, and JAK1 signaling molecule and the top 3 inhibited molecules were SOCS3, NFIL3, and NCOR1.

**Table 1:** Demonstrate the number of genes altered in response to Alpha1AR agonist phenylephrine in the middle cerebral arteries from the four developmental age groups studied.

Age Groups	Total	No. of genes upregulated in all age groups in response to Alpha1AR agonist phenylephrine	No. of genes downregulated in all age groups in response to Alpha1AR agonist phenylephrine	No. of genes differentially regulated in response to Alpha1AR agonist phenylephrine
Premature Fetus	1269	740	529	
Near-Term Fetus	1106	632	474	
Newborn Lamb	1016	614	402	
Adult Sheep	438	283	155	
Premature vs Near-Term Fetus	641	388	231	22
Near-Term Fetus vs Newborn Lamb	472	296	169	7
Newborn Lamb vs Adult Sheep	361	243	116	2
All Four Age Group Comparison	213	153	55	5

### Differential gene expression in response to Alpha1AR stimulation in near-term fetus versus newborn lamb

Birth is a major stressor for the fetus. Following birth, several hemodynamic changes occurs, such as the closure of ductus arteriosus and increased perfusion of lungs, increase in oxygen saturation of blood, etcetera. The present study gave us an opportunity to examine differential gene regulation following birth in MCAs following Alpha1AR stimulation. As demonstrated in the Table 1, on stimulation of Alpha1AR 1106 and 1016 were significantly altered in the near-term fetus and the newborn lamb MCAs, respectively. Of these altered genes, 472 were common in both age groups. Out of these 472 genes commonly altered, 296 were upregulated in both the age groups, 169 were downregulated, and 7 were altered in opposite direction (Supplemental Table S8). Furthermore, 634 genes were only altered in the near-term fetus MCA and not in the newborn lamb MCA on exposure to Alpha1AR agonist (Supplemental Table S8). Similarly, 544 genes were exclusively altered in the newborn lamb MCA and not in the near-term fetus MCA on exposure to Alpha1AR agonist (Supplemental Table S8). We conducted the pathway analysis on the genes commonly altered in the near-term fetus and the newborn lamb MCA, as well as on those which were specifically altered in either near-term fetuses or newborn lambs. A total of 38 pathways were altered commonly in both the near-term fetus and the newborn lamb MCAs in response to Alpha1AR stimulation. Of these 38 pathways commonly altered, 37 were activated and one pathway was inactivated on stimulation with Alpha1AR at both developmental stages (Supplemental Table S9). Of note, a total of 36 pathways were altered exclusively in the near-term fetus and not in the newborn lamb MCA on stimulation of Alpha1AR. All these pathways were found to be activated and none of the pathways was identified to be in the inactivated state (Supplemental Table S9). Similarly, we examined the pathways which are altered only in the newborn lamb MCAs on stimulation with Alpha1AR and not in those from the premature fetus. We identified 15 pathways which were activated in a newborn lamb’s MCA on stimulation with Alpha1AR, which were not activated in MCA from the near-term fetus. Moreover, two pathways were identified to be in the inactivated state in the newborn lamb’s MCA on stimulation with Alpha1AR, as compared to those from the near-term fetus (Supplemental Table S9). The upstream regulator analysis identified 28 molecules commonly altered by Alpha1AR stimulation in both near-term fetus and newborn MCA. Out of these 28, 26 were activated and 2 were identified to be in inactivated state (Supplemental Table S10). Of note, on comparing the upstream regulators in the near-term fetus versus the newborn lamb, we identified 19 regulators to be activated and 9 regulators to be in the inactivated state only in the near-term fetus MCA on stimulation with Alpha1AR agonist and not in those from the newborn lamb (Supplemental Table S10). Similarly, we identified 43 upstream regulators activated and 9 upstream regulators inactivated exclusively in the newborn lamb MCA on stimulation with Alpha1AR agonist as compared to MCA from the near-term fetus (Supplemental Table S10).

### Differential gene expression in response to Alpha1AR stimulation in the newborn lamb versus the adult sheep MCA

As demonstrated in the Table 1, on stimulation of Alpha1AR 1016 and 438 were significantly altered in the newborn lamb and the adult sheep MCA, respectively. Of these, 361 genes were common in both age groups. Out of these 361 genes commonly altered, 243 were upregulated in both the age groups, 116 were downregulated, and 2 genes were altered in opposite direction (Supplemental Table S11). Of note, 655 genes were altered (369 were upregulated and 286 were downregulated) in the newborn lamb MCA on stimulation with PHE, which did not show a change in expression in the adult MCA. Similarly, on stimulation with PHE we identified 77 genes which were altered (40 upregulated and 37 downregulated) specifically in the adult MCA and not in the newborn MCA. We conducted the pathway analysis on the genes commonly altered in the newborn lamb and the adult sheep, as well as on those which were specifically altered in either newborn lamb or the adult sheep. A total of 15 pathways were altered commonly in both the newborn lamb and the adult sheep MCAs in response to Alpha1AR stimulation. Of these 15 pathways commonly altered, 13 were activated and 2 pathways were inactivated on stimulation with Alpha1AR at both developmental stages (Supplemental Table S12). Furthermore, a total of 41 pathways were altered exclusively in the newborn lamb MCA and not in the adult sheep MCA on stimulation of Alpha1AR. Of these pathways, 40 were found to be activated and 1 of the pathway was identified to be in the inactivated state (Supplemental Table S12). Similarly, we examined the pathways which are altered only in the adult sheep MCA on stimulation with Alpha1AR and not in those from the newborn lamb MCA. We identified 2 pathways which were activated in the adult sheep’s MCA on stimulation with Alpha1AR, which were not activated in the MCA from the newborn lamb. Moreover, 1 pathway was identified to be in the inactivated state in the adult sheep MCA (Supplemental Table S12). The upstream regulator analysis identified 19 molecules commonly altered by Alpha1AR stimulation in both the newborn lamb and the adult sheep MCA. Out of these 19, 18 were activated and 1 was identified to be in the inactivated state (Supplemental Table S13). On further comparing the upstream regulators in the newborn lamb versus the adult sheep, we identified 68 regulators to be activated and 11 regulators to be in the inactivated state only in the newborn lamb MCA on stimulation with Alpha1AR agonist and not in those from adult (Supplemental Table S13). Similarly, we identified 4 upstream regulators activated and 4 upstream regulators inactivated exclusively in the adult MCA on stimulation with Alpha1AR agonist as compared to the MCA from the newborn lamb (Supplemental Table S13).

**Figure 7:**
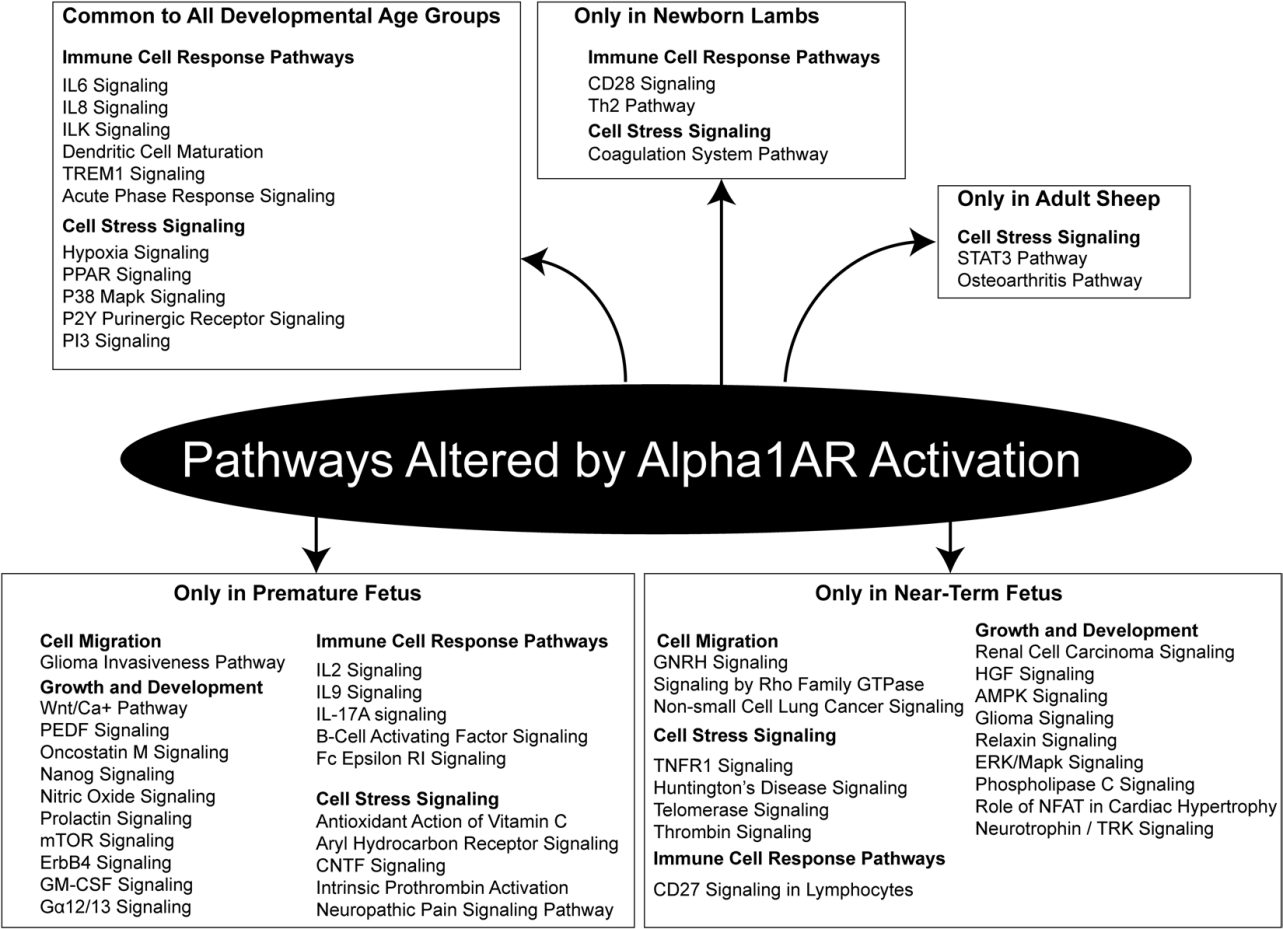
Overall schematic demonstrating pathways altered commonly in all four groups and in age specific manner.

Overall, there were pathways which were commonly regulated by Alpha1AR activation (Fig. 7) in all the developmental age groups as well as pathways which were activated in developmental age specific manner (Fig. 7).

## Discussion

Alpha1ARs are the major regulator of norepinephrine-mediated vascular regulation^28,38,57^. With developmental maturation, the adrenergic system undergoes significant changes, which play an important role in CBF regulation and cellular/vascular growth including angiogenesis^21,22,26,38,57,59–61^. In our previous studies, we have demonstrated that the premature fetus lacks the sympathetic system-mediated regulation of CBF^26^. Also, we have demonstrated that with development from the premature fetus to the adulthood, there is a substantial increase in Alpha1AR-mediated contractile responses^58^. In addition, we demonstrated that Alpha1AR expression is significantly higher in the adult sheep MCA as compared to those in the fetus^38^. In the present study, we demonstrate that Alpha1AR leads to differential gene expression in premature versus mature fetus MCAs. Also, we demonstrate that the Alpha1AR–mediated changes in gene expression further continues with development until the adulthood. Of note, the changes in gene expression in response to Alpha1AR stimulation if highest in the premature fetus MCA and decreases with development (Table 1).

Of note, Alpha1AR receptors are well known to mediate both contractile and trophic effects^21,28,43,62^ and have been shown to induce adventitial, smooth muscle, and endothelial cell growth^21,22^. On examining the genetic pathways altered by Alpha1AR stimulation irrespective of the developmental stage, there was activation of immune and inflammatory response pathways. We also found previous reports demonstrating Alpha1AR-mediated increased expression of IL6 in fibroblasts and cardiomyocytes^63–65^. These reports suggest that left ventricular hypertrophy induced by stress is mediated by the norepinephrine-IL6 pathway. However, its role in MCAs is not known. Moreover, there are other studies which show similar findings of upregulation of IL8, IL6, VEGF following sympathetic stimulation^66,67^. Moreover, regulation of other pathways identified by the present study such as ILK signaling and TREM1 signaling by Alpha1AR are not well studied. In the present study, we observed that Alpha1AR inhibited the PPAR pathway. Similar to this finding, a study has reported inhibition of PPAR expression in adipocytes following stimulation with norepinephrine^68^. However, this report suggested that the effect of norepinephrine was mediated by beta-adrenergic receptors in the adipocytes. In the present study, we used Alpha-1AR specific agonist PHE and observed a similar inhibition of PPAR expression in the MCA. Specifically, on examining pathways specific to fetal age group, Alpha1AR activation is involved in pathways regulating cellular and organismal growth (Fig. 7). However, with maturation to adult life, Alpha1AR –mediated activation of these pathways was not observed. Thus, it appears that with developmental maturation, Alpha1AR -mediated activation of pathways is altered; mechanisms of these shifts are not known, however.

As mentioned above, there was a significant overlap of genes which were altered by Alpha1AR stimulation during all the age groups studied. However, there were also an age specific signature of Alpha1AR stimulation. Studies have also demonstrated that Alpha1AR are present in premature fetus, however, they are not contributing towards regulation of CBF^26^. We speculate that during premature stage of fetus development, these receptors may be involved more in trophic functions. The pathways which were specifically activated in the premature fetus MCA and not in those from mature fetus were Tec Kinase Signaling, iCOS-iCOSL signaling, PKCθ signaling. Tec Kinases belong to tyrosine kinase and are known to be present in endothelial cells^69^ and chiefly involved in angiogenesis and vascular development as well as remodeling^70^. However, its regulation through Alpha1AR is not known. Similarly, iCOS-iCOSL signaling pathway and PKCθ signaling is known to play a critical role in angiogenesis^71,72^. Thus, it appears that in premature fetus, Alpha1AR is regulating vascular development, remodeling and angiogenesis. The top inhibited pathway specific for the premature MCA following stimulation Alpha1AR was LXR/RXR Activation pathway, which has anti-inflammatory action. Thus, the proinflammatory pathways are activated and anti-inflammatory pathways are inhibited by Alpha1AR stimulation. We also examined the pathways which were differentially altered in a mature fetus MCA as compared to those in the immature fetus. The top pathway was HGF Signaling Pathway which was specifically activated in mature ovine fetus MCA following Alpha1AR stimulation. The HGF signaling pathway has been shown to be involved in intracranial aneurysms^73^. The other pathways involved were AMPK, CD27, Telomerase Signaling and so forth. Most of these pathways are known to play a significant role in organismal development and growth, however, their role in cerebrovascular development requires further investigation.

In the present study, we also observed significant alterations in the gene expression with birth. As well established, birth is associated with several changes in the blood circulation. With birth, the placental circulation is abolished and lung takes the role of oxygenation. The oxygen saturation changes from ~30 mm Hg^74^ to ~75 mm HG^75^. There is a significant increase in blood pressure with birth^76^ and CBF is regulated accordingly^77^. Importantly, with birth there is a significant increase in norepinephrine levels in both human^78,79^ and sheep^80,81^. Of importance, we identified several genes (Supplemental Table S7) pathways (Supplemental Table S9), and upstream regulators (Supplemental Table S10) which were altered in newborn lamb MCA on stimulation with Alpha1AR and not in near-term fetus. Notably, several pathways which were altered in premature fetus and not in near-term fetus are re-activated again following birth. We also observed this U-Shaped regulation of gene/pathway expression in our previous studies^51^. The pathways which are activated during premature fetus life and postnatal life are important in vascular growth and development.

During adult life, norepinephrine is known to produce arterial contractility through Alpha1ARs^38,57^. Moreover, we have demonstrated that Alpha1AR–mediated contractile response increases with maturation^58^. Also, Alpha1AR expression is significantly higher in the adult sheep MCA as compared to those in the fetus^38^. The alpha-adrenergic signaling pathway is not only the chief regulators of cerebrovascular tone and CBF but plays an important role in angiogenesis^21,22,28,59,82^. In the present study, we observed a significant upregulation of VEGF and other genes involved in angiogenesis on stimulation with Alpha1AR agonist. On comparison of the adult sheep MCA to the newborn lamb MCA, we observed only three pathways which were altered exclusively in the adult MCA, these were osteoarthritis signaling pathway, TNFR1 signaling pathway, and STAT3 pathway. The top gene which was altered in the adult MCA but not in the newborn lamb MCA was Fos gene. Similar to the finding in the present study, it has demonstrated in the past that norepinephrine can lead to increase in Fos expression in the heart^83^. Moreover, adrenergic system-mediated activation of the STAT3 pathway has been demonstrated to be involved in angiogenesis^84^. Overall, we observed a reduction in the number of genes altered by Alpha1AR stimulation from premature fetus to adult sheep MCA.

Alpha1AR not only produce vasoconstriction, but also plays an important role in vascular development, remodeling, and angiogenesis. However, at present, there have been few studies demonstrating its effect on gene expression. This is the first study to examine Alpha1AR effect on genetic pathways with organismal development in the cerebral artery. With maturational development, there is a significant increase in norepinephrine levels and expression of Alpha1ARs. However, the changes in downstream molecules are not well studied. In the present study, we demonstrated a pool of gene which is altered in the cerebral arteries irrespective of the age group analyzed. At the same time, we demonstrate that Alpha1AR stimulation produces age-specific changes in gene expression from a premature fetus to a mature fetus to a newborn to an adult organism. Many premature babies have problems in the regulation of blood flow to their brains, which may be, in part, a consequence of immature sympathetic regulation. This dysregulation may have serious consequences with intraventricular and germinal matrix hemorrhage, with long-term neurological sequelae. The present study identifies several important genes, pathways, and upstream regulators acting differently in response adrenergic stimulation in different age groups. However, further investigation is needed to identify specific molecules, which makes fetus vessels more vulnerable to dysregulation.

## Electronic supplementary material

S1

S2

S3

S4

S5

S6

S7

S8

S9

S10

S11

S12

S13
